# Compression syndromes of the popliteal artery due to intramuscular ganglion cyst of the biceps femoris: A case report and literature review

**DOI:** 10.3389/fcvm.2022.1018694

**Published:** 2022-11-25

**Authors:** Kai Zheng, Wentao Yang, Hualiang Ren, Shengxing Wang, Mingsheng Sun, Wangde Zhang, Chunmin Li

**Affiliations:** Department of Vascular Surgery, Beijing Chaoyang Hospital, Capital Medical University, Beijing, China

**Keywords:** ganglion cyst of knee joint, intramuscular (IM), biceps femoris long head, popliteal artery (PA), entrapment syndrome

## Abstract

Intramuscular ganglion cyst (IMGC) is a very rare lesion with an unidentified pathogeny that originates within the muscle. We encountered a case of 49-year-old man who complained of intermittent claudication in the right lower limb for 2 months. An intramuscular ganglion cyst in the biceps femoris muscle was diagnosed and located by Computed tomography angiography (CTA) and magnetic resonance imaging (MRI), which compressed the popliteal artery and resulted in ischemia in the right lower limb. Six months after surgical resection, there was no recurrence of the cyst and the popliteal artery was patency. We describe this case with a review of the relevant literature.

## Introduction

A ganglion cyst is a common tumor-like lesion arising from various soft tissues that is generated by mucoid degeneration of the joint capsule, tendon, or tendon sheath (1). It usually occurs near the joints and ligaments of the extremities, especially the dorsal wrists and ankles, and occurs in young women between 20 and 40 years old (2, 3). However, an intramuscular ganglion cyst which originates within the muscle belly itself and generates symptoms of arterial compression concomitantly is a rare lesion (4, 5). As previously reported, diagnosis, localization, and complete resection of intramuscular tendon sheath cysts are challenging, and surgical exploration and pathological diagnosis are also necessary (6). Computed tomography angiography (CTA) and magnetic resonance imaging (MRI) can distinguish ganglion cysts from other soft tissue tumors and tumor-like lesions and provide particular information to determine the location of lesions. As far as we know, this is the first case report of an intramuscular ganglion cyst arising from the biceps femoris.

## Case presentation

A 49-year-old male was admitted to hospital with intermittent claudication of the right lower limb for 2 months, with a limping distance of 100 m and no resting pain (Rutherford III), which is an indication for surgery (7). His past medical history includes coronary artery disease and hyperlipidemia.

Physical examination revealed that the popliteal, dorsalis pedis, and posterior tibial arteries were not observed. His skin color, skin temperature, and lower limb appearance were basically normal, and the capillary filling time of the bilateral toes was < 2 s.

**FIGURE 1:**
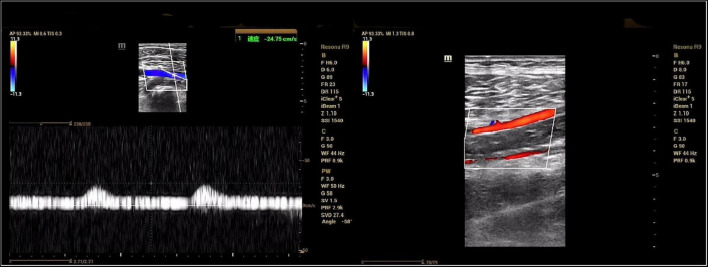
Preoperative ultrasonography. The spectral pattern was changed and triphasic waves were absent.

**FIGURE 2:**
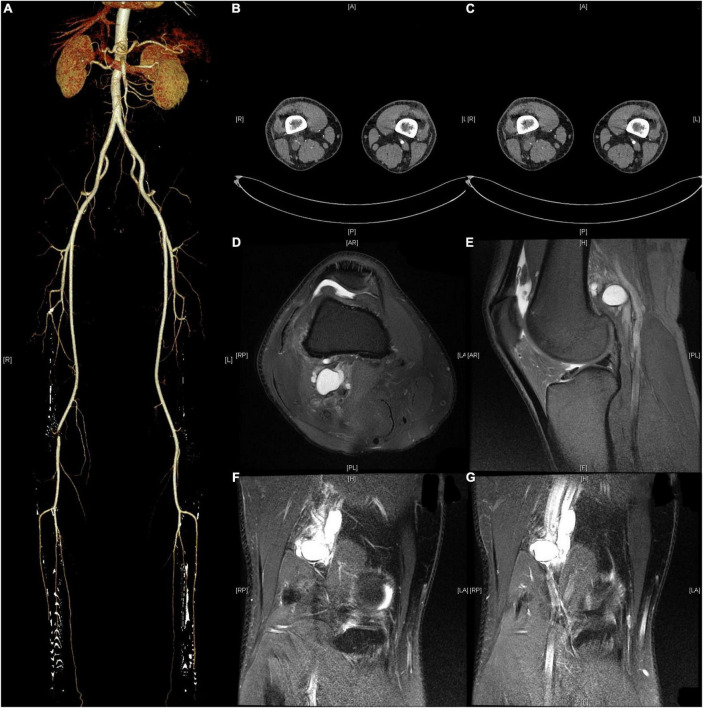
Preoperative CTA and MRI. **(A–C)** CTA showed severe stenosis of the popliteal artery in the right lower extremity. **(D–G)** MRI suggested that the popliteal artery was compressed by an oval cyst with high signal intensity on T2-weighted, which is 1.7*2.5*3.1 cm in volume.

Ultrasound showed reduced flow velocity in the right lower limb artery, superficial femoral artery (34 cm/s), popliteal artery (24 cm/s), anterior tibial artery (20 cm/s), posterior tibial artery (17 cm/s), and peroneal artery (24 cm/s), with absent triphasic waves (Figure 1). CTA suggests severe stenosis and almost complete occlusion of the P1 segment of the popliteal artery (Figures 2A–C). MRI suggested that the popliteal artery was compressed by an oval cyst with high signal intensity on T2-weighted, which is 1.7*2.5*3.1 cm in volume (Figures 2D–G). A diagnosis of popliteal artery entrapment syndrome is proposed.

**FIGURE 3:**
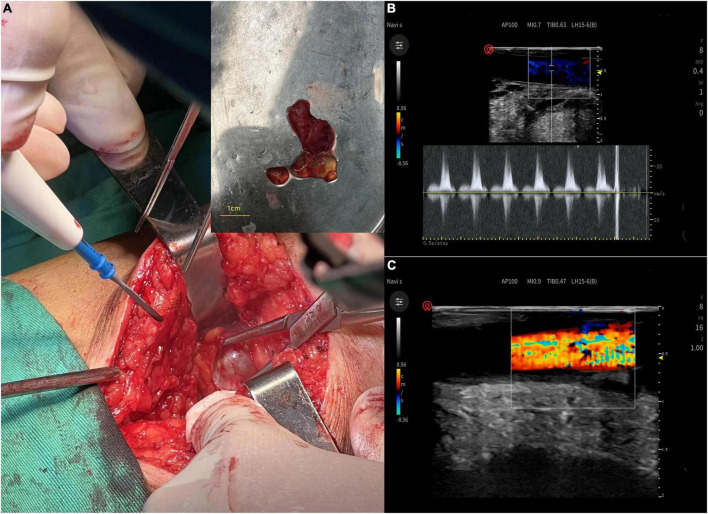
**(A)** The popliteal artery was connected to a tendon sheath cyst on the medial aspect of the long distal head of the biceps femoris tendon with a smooth, tough wall, and jelly-like contents. **(B,C)** After cyst removal, blood flow of popliteal artery and triphasic waves were restored.

**FIGURE 4:**
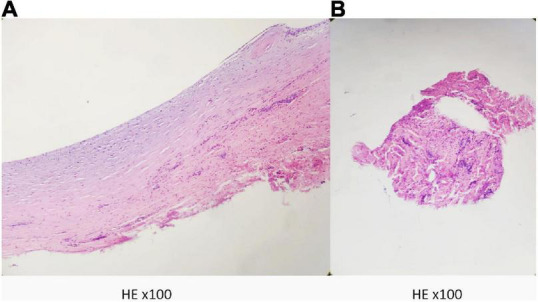
Postoperative pathology suggests a fibrous cyst wall. **(A)** The wall of the cyst. **(B)** The contents of the cyst.

Exploration of the right popliteal artery and cystectomy were performed under epidural anesthesia. The popliteal artery was connected to a tendon sheath cyst on the medial aspect of the long distal head of the biceps femoris tendon with a smooth, tough wall, and jelly-like contents (Figure 3A). After cyst removal, blood flow of popliteal artery and triphasic waves were restored (Figures 3B,C). Postoperative pathology suggests a ganglion cyst (Figure 4).

At the 6-month postoperative follow-up, the patient had no intermittent claudication, good arterial pulsation in the lower limbs and no significant ultrasound abnormalities.

## Discussion

Ganglion cyst (GC) is a common and frequent clinical condition, but cases of tendon cysts compressing the surrounding blood vessels are rarely reported. Ganglion cysts that occur within the muscle belly and are associated with popliteal artery compression are extremely rare. Limited data available make them prone to misdiagnosis and underdiagnosis, and preoperative imaging to determine their origin and nature can be difficult. Moreover, previous studies were unable to generate valuable research to develop a uniform and effective treatment due to the small number of cases included (6, 8–14).

Entrapment neuropathy is a frequent clinical symptom because of its anatomical location. Also, the popliteal vascular components are subject to compression, including the vein, which is next most medially located and easily compressible compared with the artery, which is most lateral and least frequently involved (15). The popliteal artery is deeper and stiffer than the vein and requires greater force to produce compression, so the incidence of this syndrome is minimal. Only in rare cases, when the popliteal artery is compressed by the cyst in a pulsatile manner, the patient may develop lower limb claudication due to intermittent ischemia of the lower limb (16–20). Venous compression is not mentioned in reported cases of arterial occlusion, but the vein may also be compressed and ignored because the symptoms of arterial compression are of more clinical importance. After the removal of cysts, the pressure on any of the blood vessels is relieved.

The primary differential diagnosis is cystic adventitial disease (CAD), which typically occurs in otherwise healthy, middle aged male patients, causing symptoms of sudden-onset progressive intermittent claudication. Although four theories have been proposed, the etiology of CAD is still not fully understood (21, 22). During vascular embryonic development, undifferentiated mesenchymal cells are incorporated into the arterial wall. It is these mucin-secreting mesenchymal cells that subsequently produce mucoid material, from which epitaxial cysts arise. This hypothesis is considered to be the most reasonable explanation for CAD. Also, the etiology of intrasynovial ganglion cysts is unknown. Repeated damage to the tendon sheath with subsequent cystic changes may be the cause of intrathecal ganglion cysts, as tenosynovitis or associated tendon tears are often seen around ganglion cysts (9). During the operation, it was clear that the cyst originated from the muscle belly rather than the popliteal artery, supporting the final diagnosis.

There is controversy regarding the final diagnosis of the disease. Popliteal artery depression syndrome (PAES) is defined as a group of symptoms in which there is a congenital anatomical abnormality between the popliteal artery and its surrounding muscles or bundles of tendons and fibrous tissues (23). Classification of PAES, currently used and widely accepted, was proposed by Love and Whelan (24) and revised by Rich et al. (25). In terms of this system, type I is associated with an aberrant medial arterial course around the normal medial head of the gastrocnemius muscle. In type II, an abnormal medial head of the gastrocnemius inserts laterally on the distal femur and displaces the popliteal artery medially. Type III is depicted by an aberrant accessory slip from the medial head of the gastrocnemius muscle that wraps around the popliteal artery. In type IV, the popliteal artery is entrapped by the popliteus muscle. In type V, the popliteal vein is also involved. Type VI is considered functional and is recognized in the presence of a normally positioned popliteal artery that is entrapped by a normally positioned but hypertrophied gastrocnemius muscle. Characteristics of this patient do not conform to any types of PAES.

Popliteal cysts are also known as Baker cysts, a general term for synovial fluid cysts in the popliteal fossa that occur in the medial head of the semimembranosus bursa (gastrocnemio-semimembranosus bursa, GSB). Secondary popliteal cysts are most often seen in adults and the cysts tend to be connected to the knee joint cavity. Sansone et al. performed a retrospective analysis of 1,001 cases of MRI for various reasons and found popliteal cysts in 4.7–37% of these cases, all of which communicated with the joint cavity. The patient in this case originated within the biceps femoris muscle belly and was not a synovial bursa of the medial head of the semimembranosus and gastrocnemius muscles and did not communicate with the joint cavity and was not strictly a popliteal cyst (26).

Several treatment options are available if the diagnosis of PAES has been made, with the treatment objective being to release the popliteal artery from compression and preserve popliteal arterial flow (27). Conventional surgery, endovascular surgery, thrombolysis, or a combination of these modalities are all reasonable treatment options depending on the patient’s clinical symptomology and anatomy (28). If the artery is occluded, stenotic, or aneurysmal, vascular reconstruction is mandatory in addition to the division of any entrapping structure (29). In this patient, after intraoperative resection of the cyst, the popliteal artery blood flow was confirmed by ultrasound, and no further vascular repair or autograft of the great saphenous vein was performed.

## Conclusion

Here we describe a patient with an IMGC of the biceps femoris muscle compressing the popliteal artery, which could not be diagnosed preoperatively and highlighted the necessity and difficulty of differential diagnosis. Intraoperative exploration and postoperative histopathology are key to the diagnosis of IMGC. In our case, intraoperative ultrasound did not reveal any abnormality in the popliteal artery and there were no clinical symptoms at the 6-month follow-up, and the follow-up ultrasound was normal. In cases where the popliteal artery has been compressed for a short period of time and where there is no thrombosis, intimal thickening, or aneurysm formation, intraoperative ultrasound can be used to determine the flow velocity and flow in the popliteal artery, and if the flow is normal, decompression of the popliteal artery alone can be performed without wall repair or saphenous vein grafting.

## Data availability statement

The original contributions presented in this study are included in the article/supplementary material, further inquiries can be directed to the corresponding authors.

## Ethics statement

Written informed consent was obtained from the participant/s for the publication of this case report. Written informed consent was obtained from the individual(s) for the publication of any potentially identifiable images or data included in this article.

## Author contributions

KZ, WY, WZ, and CL contributed to conception and design of the study. KZ wrote the first draft of the manuscript and drew the illustrative figures. WY, HR, SW, and MS had the acquisition, analysis or interpretation of clinical data for the work. All authors contributed to manuscript revision, read, and approved the submitted version.
